# The self-care situation analysis of reproductive-aged women in Tehran: a survey study

**DOI:** 10.1186/s12905-023-02763-9

**Published:** 2023-11-25

**Authors:** Masoumeh Simbar, Zahra Kiani, Zeinab Nasiri, Nahid KhodaKarami, Soheila Nazarpour, Farzaneh Rashidi Fakari, Sepideh Keyvanfar, Hamid Alavi Majd

**Affiliations:** 1 Midwifery and Reproductive Health Research Center, Shahid Beheshti University of Medical Sciences, Tehran, Iran; 2 Department of Midwifery and Reproductive Health, School of Nursing and Midwifery, Shahid Beheshti University of Medical Sciences, Tehran, Iran; 3 General Directorate of Health, The Deputy of Social and Cultural Affairs of Tehran Municipality, Tehran, Iran; 4 Men’s Health and Reproductive Health Research Center, Shahid Beheshti University of Medical Sciences, Tehran, Iran; 5 Department of Midwifery, Chalous Branch, Islamic Azad University, Chalous, Iran; 6 Department of Midwifery, School of Medicine, North Khorasan University of Medical Sciences, Bojnurd, Iran; 7 Department of Biostatistics, School of Paramedicine, Shahid Beheshti University of Medical Sciences, Tehran, Iran

**Keywords:** Self-care, women’s health, Reproductive health, Iran

## Abstract

**Background:**

Along with a global increase in the prevalence of infectious and non-communicable diseases, self-care with an emphasis on reproductive health Self-care has received special attention. Given the importance of women’s health, assessment of their self-care status using a valid and reliable tool seems to be necessary to determine the needs for future women’s reproductive health promotion interventions. The present study aimed to assess the women’s self-care at reproductive age in Tehran, to determine women’s health needs based on global guidelines for women’s health.

**Methods:**

This was a descriptive cross-sectional study on 1051 women of reproductive age, living in Tehran. The Subjects were recruited using a multi-stage sampling method. The women completed a socio-demographic and valid and reliable questionnaire to assess their self-care status. The data were analyzed using SPSS 24 and by Pearson, Spearman, ANOVA, and regression tests.

**Results:**

The mean score of self-care was 49.57 ± 23.50% in the reproductive-aged women. The lowest scores were related to psychosocial health (32.12 ± 29.93%) and reproductive-sexual health (49.74 ± 27.99%) respectively. There were significant positive correlations between the self-care and women’s education level (*r* = 0.180; *p* < 0.01), and husband’s education level (*r* = 0.272; *p* < 0.01), while there was a negative significant correlation between the self-care and the family size (*r* = - 0.135; *p* < 0.01).

**Conclusion:**

The findings showed inadequate self-care among reproductive-aged women in Tehran. The most important challenge in their self-care behaviors was related to psychosocial and reproductive-sexual health. It seems to provide a package for promoting women’s self-care in four areas of physical, psychosocial, reproductive-sexual health, and screening tests, with an emphasis on the first two priorities, namely psychosocial and reproductive health necessary in Tehran.

**Supplementary Information:**

The online version contains supplementary material available at 10.1186/s12905-023-02763-9.

## Plain Language Summary

The framework for integrated, people-centered health services was introduced in 2016 by the World Health Organization (WHO) (World Health Organization, Interim report: placing people and communities at the Centre of health services: WHO global strategy on integrated peoplecentred health services 2016-2026: executive summary, 2015). A people-centered approach supports health literacy so that people can take responsibility for their health with evidence-based self-care interventions. When people have agency and autonomy, they can make and enact decisions in all aspects of their lives, including health (World Health Organization, WHO consolidated guideline on self-care interventions for health: sexual and reproductive health and rights: executive summary, 2019). The WHO defines self-care as “the ability of individuals, families, and communities to promote health, prevent disease, maintain health, and to cope with illness and disability with or without the support of a healthcare provider”. Self-care allows people to become agents of their health, leading to improved outcomes, especially in the area of sexual and reproductive health where stigma may prevent them from seeking care. Women and girls can identify their own health needs, and effectively manage their health conditions through self-care approaches. The WHO places great emphasis on promoting self-care, especially in women’s reproductive-sexual health, and encourages countries to design and implement their programs. Given the diversity of women’s self-care needs, this study designed women’s self-care assessment tools in two reproductive age groups in 4 dimensions including physical, psychosocial, sexual-reproductive health, and screening tests, based on a review of the valid international guidelines. The results showed that women are practicing only half of self-care and the main challenges were in psychosocial health and sexual-reproductive-health self-care in Tehran. It seems that evidence-based planning is necessary to promote self-care among reproductive-aged women.

## Background

Self-care means “the ability of individuals, families, and communities to promote health, prevent disease, maintain health, and cope with illness and disability with or without the support of a healthcare provider” [1]. The examples for self-care are health promotion, disease prevention, self-medication, seeking care, and rehabilitation [2]. Self-care is a critical path for promoting health [3].

Self-care is crucial in all aspects of health, including physical, psychosocial, reproductive, and sexual health, along with periodic screening and testing [4]. Since women are more vulnerable than men to the reproductive ill condition, a special emphasis is placed on women’s health in the Global Strategy for the Health of Women [5, 6], especially in their reproductive years [7]. Reproductive health self-care means that women identify their health needs and seek sexual-reproductive health services [2]. The most important dimensions of self-care in reproductive health include women’s physical, psychological, and social health, family planning, infertility, abortion, sexually transmitted diseases and AIDS, reproductive system cancers, gynecological diseases, and violence [8].

The WHO strives for all individuals to benefit from people-centered reproductive health services with an emphasis on women’s self-care promotion [9, 10]. Promoting women’s self-care in reproductive health is essential since many women are unable to exercise autonomy over their bodies and are unable to make decisions about their sexual reproductive life [4, 11]. SRH is one of the most significant concerns in health care, as a large number of women suffer from various sexually transmitted infections (STIs), unwanted pregnancies, abortion, domestic violence, and sexual abuse [11, 12].

The requirement for any intervention is a needs assessment based on the standards. The Standards for women’s self-care in SRH are provided in the guidelines or by reputable institutions such as the College of Obstetricians and Gynecologists or the Association of Women’s Cancer [13–15]. These guidelines can serve as the basis for a needs assessment and planning of women’s self-care programs. The WHO acknowledges that there is no standard tool for assessing the needs of women’s self-care in SRH, so it seems that developing a valid and reliable tool is crucial. This study aimed to assess the needs of reproductive-aged women’s self-care in different regions and neighborhoods of Tehran.

## Methods

### Study design

This was a descriptive cross-sectional study.

### The participants

The participants were 1051 reproductive-aged women (including 536 women aged 19-39 and 515 women aged 40-55 years) living in all 22 districts of Tehran in 2021.

The inclusion criteria were: resident of Tehran, having no known medical condition, and having an appropriate mental state and communication to fill out the questionnaire. The exclusion criteria were incorrect completion of the self-report questionnaire.

### Sample size

Sampling was performed in all 22 districts of Tehran through the health houses of the Municipality and using a multi-stage sampling method by an online questionnaire (google form). The link to the questionnaire was sent by the head of the health house who had access to the participants’ cellphone numbers. The sample size was obtained by using the following formula to calculate the sample size for each group of 19-39 and 40-55 years-old women.

$$n\ge \frac{z_{1-\alpha /2}^2\left(1-P\right)}{\varepsilon^2P}$$

The minimum sample size was calculated at 385 samples for each group using the formula of descriptive studies and considering the 50% probability of women’s self-care and the Type I error of 0.05 and the absolute error of 0.5.

### Sampling method

The sampling method of the study was similar to our previous study to assess postmenopausal women’s self-care needs [16]. A multi-stage sampling method was used to recruit the participants of the study. All 22 districts of the municipality of Tehran were selected as the clusters for sampling. Then, simple random sampling method using Excel software was used to select three or four health houses in each district.

Thereafter, using the quota sampling method and based on the population covered by the center the sample size for each health house was considered 10 to 15 eligible women. Afterward, the link to the online questionnaire was sent to the participants following contacting them and explaining the goals and process of the study and obtaining oral consent. Then electronic written informed consent was also obtained from all participants and completing the forms was only possible after giving the informed consent of the participant. For illiterate women, the questionnaire was filled out by the head of the health center through a telephone interview. The sampling of this study was accomplished during the pandemic of Covid-19.

Instructions for the recruitment of the sample were provided to the heads of the selected municipality health houses. Thereafter, an online workshop was conducted by the main researcher to train head of health houses about the procedure of the sampling. Then the head of each health house selects 10 to 15 eligible reproductive-aged women. The contact number of the main researcher was also provided to the research colleagues to answer the possible questions of the colleagues.

### Tools for data collection

This online Google form comprised two questionnaires for data collection, including (1) socio-demographic questionnaires and (2) a questionnaire to assess Reproductive-aged Women’s Health Self Care (RWSCQ) with 36 questions for 19-55 years old women.

1. The socio-demographic questionnaire: This questionnaire contained 15 questions about the personal, social, economic, and anthropometric characteristics of participants including, district, age, weight, height, education, and occupation of women, marital status, employment and education of the spouse if married, adequacy of income, housing status, number of children, medical history and condition.
2. Reproductive-aged Women Health Self Care (RWSCQ): This questionnaire was developed with 36 items in 4 domains including the physical health domain with 14 items, psychosocial health with 6 items, and reproductive-sexual health with 12 items, and periodic tests with 4 items. The questionnaire assessed the self-care of 19-55 years old women.

This questionnaire was developed using a deductive approach and based on a review of the guidelines for women’s health, reproductive health, and self-care which were presented on the site of trustworthy organizations such as *the American College of Obstetricians and Gynecologists (ACOG)* [17]*, Royal College of Obstetricians and Gynecologists* [18]*,* Medline Plus [19], World Health Organization women’s health [20], Centers for Disease Control and Prevention (CDC) [21]. Finally, the items of the primary questionnaire were generated based on the updated Women’s Preventive Services Initiatives WPSI “2021 recommendations for well-woman care” [22, 23]. This chart is adapted by the members of the advisory panel support for the WPSI including, ACOG, the American Academy of Family Physicians, and the American College of Physicians (ASP). The items were selected and modified to be appropriate for reproductive-aged women and based on the guidelines of the above-mentioned reputable organizations.

To assess the validity and reliability of the questionnaire the method described by Pilot and Beck 2010 was used [24]. RWSCQ was evaluated in two age groups 19 to 39 years and 40 to 55 years. First, face validity (qualitative and quantitative) and then content validity (qualitative and quantitative) were examined.

#### The face validity

For qualitative face validity assessment, 5 reproductive-aged women were asked about the items’ difficulty, irrelevancy, and ambiguity. Afterward, the impact score of each item was calculated and evaluated by the cut-off point of > 1.5. The Impact Score was calculated by the following formula.

$$\textrm{Impact}\ \textrm{score}=\textrm{frequency}\ \left(\%\right)\times \textrm{importance}$$

All items of the questionnaire had a score of more than 1.5 and so were considered important by the participants. The calculated Impact scores of RWSCQ were 2.47 to 4.86.

#### The content validity

Content validity of the questionnaire was assessed by 12 experts in midwifery, public health, reproductive health, and nurses. The content Validity of the questionnaire was assessed by calculating Content Validity Ratio (CVR) and Content Validity Index (CVI). The results showed CVR ranged from 0.83 to 1. The modified content validity index of I-CVI for all items ranged from 0.91 to 1, and the S-CVI / Ave score was 0.97. An alpha coefficient above 0.7 is usually acceptable [25].

#### The reliability

The reliability of the questionnaire was measured by calculating Cronbach’s alpha coefficient for internal consistency assessment and also calculating the Pearson coefficient to measure the stability of the questionnaire by the test-retest method on 15 reproductive-aged women. The results showed the reliability of the questionnaire by *a* = 0.92, and Intra-class Correlation Coefficient ICC = 0.93. A reliability coefficient higher than 0.7 is acceptable [26].

#### The scoring

The items were scored 1 to 3 in the responses of “No, I did not”, “Yes, somewhat/ I intend to do” and “Yes I did”, respectively. The score ranges for the different dimensions of the questionnaire including physical-, psychosocial-, and sexual-reproductive health and screening tests were 14 to 42, 6 to 18, 12 to 36, and 4 to 16, respectively, and for the whole questionnaire was 36-108 for RWSCQ-36. The higher scores indicate healthier self-care behaviors of reproductive-aged women. The score of each domain and the total score were calculated and then converted to the standardized 0 to 100 score using the following eq. (X-Min Score / Max-Min Score) × 100. The questionnaire is available in Additional file 1.

### Statistical analysis

After filling out the Google forms the participants in the Google platform, the data were generated in the Excel software in two Google Drive for women ages 19-39 and 40-55 years old. Then the data in the Excel file was converted to SPSS. Then, the data were analyzed using SPSS 24 and by Kolmogorov–Smirnov to test the normality of data, ANOVA, Sheffe, Pearson, and Spearman correlation coefficient tests, and linear multiple regression analysis. *P* values less than 0.05 and a confidence interval of 95% were considered statistically significant.

### Ethics

The study was approved by the ethics committee of Shahid Beheshti University of Medical Sciences, with the code “IR.SBMU.PHARMACY.REC.1398.298”. All methods were performed under the relevant guidelines and regulations as approved by the deputy of research and the ethical committee of Shahid Beheshti University of Medical Sciences. An online written informed consent was obtained from all participants.

## Results

One thousand fifty-one reproductive-aged women including 536 women aged 19- 39 years and 515 women aged 40-55 years old completed the questionnaire. The socio-demographic characteristics of women are shown in Table 1.

**Table 1:** Socio-demographic characteristics of the reproductive-aged women participated in the study, Tehran 2021 (*n* = 1051)

Variables		Mean ± SD	Minimum	Maximum
Age (years)		38.61 ± 8.76	19	55.0
BMI (Kg/m 2)		24.59 ± 3.96	16.0	41.0
Duration of marriage (years)		16.55 ± 10.03	1	42.0
Number of children		1.40 ± 1.15	0	6.0
Family size		3.47 ± 1.06	1	12.0
		N (%)		
Marital status	Single	188 (17.9)		
	Married	812 (77.3)		
	Divorced	34 (3.2)		
	Widow	17 (1.6)		
Education	Illiterate	12 (1.1)		
	Lower Diploma	72 (6.9)		
	Diploma	300 (28.5)		
	Academic	667 (63.5)		
Education of Husband	Illiterate	13 (1.5)		
	Lower Diploma	79 (9.4)		
	Diploma	291 (34.7)		
	Academic	459 (14.4)		
Occupation	Housewife	592 (56.3)		
	Employed	447 (42.5)		
	Retired	12 (1.1)		
Husband Occupation	Unemployed	36 (4.3)		
	Employed	252 (30.4)		
	Freelance	468 (56.5)		
	Retired	72 (6.9)		
Home ownership status	The owner	518 (58.8)		
	Non-owner	364 (34.6)		
	Relatives’ house	69 (6.6)		
The adequacy of family’s monthly income	Inadequate	529 (50.3)		
	Adequate	495 (47.1)		
	More than adequacy/savings	27 (2.6)		

The finding demonstrated the highest mean score of self-care for the screen tests and physical health, and the lowest score for the psychosocial health self-care among the reproductive-aged women (Table 2).

**Table 2:** The self-care status of the reproductive-aged women in different domains of health, Tehran 2021 (*n* = 1051)

Self-care Domains	Mean	SD
Physical Health	56.93	24.49
Psychosocial Health	32.12	29.93
Sexual-Reproductive Health	49.74	27.99
Periodic tests and exams^a^	69.27	30.428
Total	49.57	23.50

^a^Self-care for 40 -55 years’ women; *n* = 515

The self-care assessment of the women in the physical health domain showed the highest scores for the items of “measuring height and weight” and “Oral health and fluoride consumption” and the lowest score for “sleep disorders “and “safe use of mobile phones and the internet”.

The self-care assessment in the psychosocial health domain indicated the highest scores for “personal and family relationships” and the lowest score for “suicidal thoughts and attempts”. The reproductive-sexual health domain showed the highest level of self-care in “Menstrual health and hygiene” and the lowest score for performing the “AIDS test”. The self-care assessment of the women in the domain of periodic tests showed the highest level of self-care for the “blood lipids test” and the lowest score for the “stool test” (Table 3).

**Table 3:** The self-care status of reproductive-age women in all items of self-care, Tehran 2021 (*n* = 1051)

Self-Care	Mean	SD
Physical Health		
Height and weight measurement	2.51	0.71
Oral health and fluoride consumption	2.44	0.69
Periodic tests to prevent the risk of chronic diseases	2.40	0.75
Taking vitamins and minerals	2.39	0.74
Nutrition	2.27	0.79
Physical activity	2.17	0.79
Use of complementary and herbal medicines	2.11	0.84
life style	2.10	0.79
Safe driving	2.05	0.91
Using sunlight to absorb vitamin D.	2.04	0.81
Accident prevention	2.00	0.86
Avoid smoking, alcohol, tobacco drugs	1.92	0.93
Safe use of mobile phones and the Internet	1.90	0.85
sleep disorders	1.63	0.79
Psychosocial Health		
personal and family relationships	1.96	0.86
Anxiety and stress	1.71	0.79
Job Satisfaction	1.64	0.80
Depression	1.64	0.80
physical, sexual and emotional violence	1.46	0.74
Suicide thoughts and attempts	1.34	0.684
Sexual-Reproductive Health		
Menstrual health and hygiene	2.58	0.69
Contraception	2.23	0.87
Breast self-examination	2.23	0.84
Breast examination by health personnel	2.10	0.90
Pap smear in the last 3 years	2.08	0.91
Mammography^a^	1.97	0.91
Prevention of sexually transmitted diseases and AIDS	1.92	0.92
Screening for sexually transmitted diseases	1.81	0.88
Urinary fecal incontinence^a^	1.56	0.82
Sexual health and function	1.55	0.82
Pelvic Floor Disorders exam^a^	1.50	0.80
AIDS test	1.46	0.79
Periodic Screen Tests		
Performing blood lipid test in the last 5 years^a^	2.62	0.68
Diabetes screening in the last 3 years^a^	2.55	0.72
Performing thyroid screening in the last 5 years^a^	2.49	0.77
Stool test	1.88	0.92

^a^Self-care for 40 -55 years women; *n* = 515

**Fig. 1:**
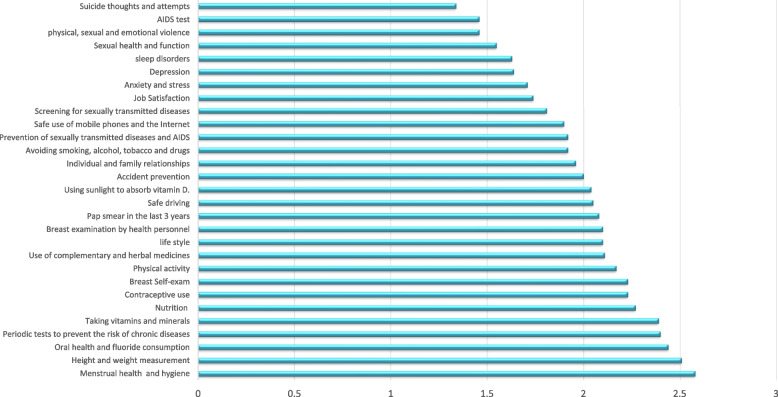
The highest to lowest scores for self-care measures in the reproductive aged women Tehran 2021 (*n* = 1051)

The self-care assessment of the women in all health domains showed the highest score for “menstrual health” and the lowest score for “suicide thoughts and attempts” (Fig. 1).

**Fig. 2:**
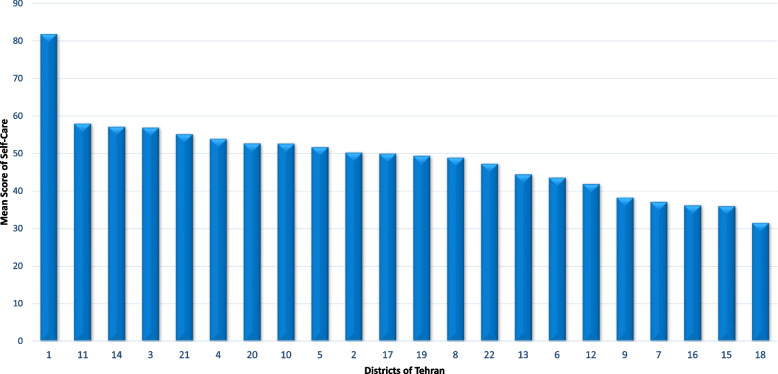
Mean scores of self-care in reproductive-aged women in 22 districts of Tehran-Iran

Analysis of variance showed that the mean scores of self-care were significantly different in districts of Tehran. The highest was found in District 1 and the lowest score was in District 18. Figure 2 shows the self-care scores in different districts of Tehran from the highest to the lowest in reproductive-aged women in Tehran (Fig. 2).

The finding showed a significant correlation between the women’s self-care and their family size (*r* = - 0.135), their income adequacy (*r* = 0.113), their education (*r* = 0.180), and their husband’s education (*r* = 0.272) (Table 4).

**Table 4:** Correlation between the reproductive-aged women’s self-care and their demographic characteristics

Characteristics	N	R	P	Test
Age	1050	0.037	0.22	Pearson
BMI	1050	0.016	0.608	Pearson
Number of Children	1028	-.008	0.810	Spearman
Family Size	1050	-0.135	0.00	Spearman
Duration of Marriage	840	-0.088	0.11	Pearson
Adequacy of Income	1050	0.113	0.00	Spearman
Home ownership status	1050	0.009	0.767	Spearman
Education	1050	0.180	0.00	Spearman
Husband education	838	0.272	0.00	Spearman

Analysis of variance also showed the significant difference between women’s self-care in different groups of marital status (*p* < 0.01), women’s occupation (*p* < 0.002), and their husbands’ occupation (*P* < 0.032) (Table 5).

**Table 5:** The Comparison self-care scores of reproductive-aged women in different groups of marital status, occupation and husbands’ occupation (*n* = 1051)

Charactristics		N	Mean	SD	T
**Marital Status**	Single	188	43.82	23.96	ANOVA: df = 3; F = 5.32; *p* < 0.01 Tukey: The difference between single and married groups *P* < 0.01
	Married	811	51.10	22.93	
	Divorced	37	47.31	25.62	
	Widow	17	44.62	31.29	
**Occupation**	Housewife	592	47.36	23.19	ANOVA: df = 2; *F* = 11.75; *p* < 0.002 Tukey: The difference between housewife with other groups *P* < 0.01
	Employed	446	52.34	23.54	
	Retired	12	55.89	26.83	
**Husband’s Occupation**	Unemployed	36	20.51	41.13	ANOVA: df = 3; *F* = 2.84;*p* < 0.032 Tukey: The difference between unemployed husband with other groups *P* < 0.05
	Employed	251	22.01	52.61	
	Freelance	468	23.83	50.64	
	Retired	72	24.28	53.30	
**Home ownership status**	The owner	617	49.64	23.68	ANOVA: df = 2; *F* = 0.063; *p* > 0.05
	Non-owner	364	49.31	23.28	
	Relatives’ house	69	50.34	23.27	

Linear regression test also showed the positive potential predictor variables for women’s self-care variables including family income, women’s education and occupation, husbands’ education and occupation, and the negative prediction of family size (Table 6).

**Table 6:** Linear regression model of predictors of the reproductive-aged women’s self-care

Model	Unstandardized Coefficients		Standardized Coefficients	t	Sig.
	B	Std. Error	Beta		
Constant	-22.118	14.350		-1.541	.124
Marital status	7.852	4.404	.062	1.783	.075
Marriage Duration (years)	.049	.153	.021	.322	.747
Number of Children	1.269	1.296	.059	.979	.328
Age (years)	.028	.169	.009	.166	.868
Body Mass Index	.291	.189	.055	1.539	.124
Family Size	-2.208	1.075	-.092	-2.054	.040
Education	4.719	1.445	.171	3.267	.001
Occupation	3.684	1.707	.081	2.158	.031
Income Adequacy	3.525	1.508	.083	2.338	.020
Home ownership Status	1.201	1.355	.032	.887	.376
Husband’s Education	4.270	1.277	.158	3.343	.001
Husband’s Occupation	3.360	1.199	.099	2.802	.005

## Discussion

This was the first study on the self-care status of reproductive-aged women. Self-care is crucial in all aspects of health, as a way to empower people in self-care, prevention, and diagnosis of diseases in the early stage [27]. The WHO introduced people-centered health services in 2016 and emphasized on promoting women’s self-care [4]. Since women are more vulnerable to ill conditions and also more responsible for providing family’s health and well-being, a special emphasis is placed on women’s health in the Global Strategy for 2016 to 2030 by the United Nations [4, 5]. It seems that evidence-based planning is necessary to promote self-care among women. The WHO recommends self-care interventions for every country and economic setting, as a critical path to reaching universal health coverage, promoting health, and keeping the world safe [28]. In this accordance, community education about self-care and taking responsibility about own and others’ health are the prerequisites for a successful implementation of the programs [29]. Therefore, this study analyzed the self-care situation of reproductive-aged women as the first step for planning evidence-based self-care programs.

The results of this study showed the lowest self-care scores in the psychosocial health dimension among the reproductive-aged women in Tehran. A study on postmenopausal women’s self-care needs and priorities in Tehran also showed the lowest score related to psychosocial health [16]. Although the rate of depression, stress, anxiety, violence, suicidal thoughts, and job dissatisfaction is high in the world, and in Tehran, this low level of the self-care among the women in Tehran seems to be due to two reasons: 1) seeking psychological counseling and treatment may be considered as a social stigma and 2) psychological counseling is from expensive services [30] while screening for psychological disorders such as depression, anxiety, stress, violence is strongly recommended by the WHO for all, especially for women [31, 32]. These disorders can be easily screened by standard questionnaires, and with appropriate inter-sectoral cooperation between health centers and psychological counseling services, referral to more specialized services could be possible if required. Psychosocial health promotion is highly recommended and essential because women are more than men at risk of psychological disorders, due to their physiologic, social, cultural, and economic status and gender stereotypes [33].

The second challenge for women’s self-care was sexual-reproductive health. In other words, the women meet less than half of the recommended criteria for their sexual-reproductive health care. A similar study about sexual self-care among Iranian women showed an average score of 70%, and the lowest score was related to self-care for preventing breast and cervical cancers [34]. This important dimension of women’s health includes items such as menstrual health and hygiene; fertility planning; screening to prevent gynecological cancers such as breast and cervical cancer, screening to prevent sexually transmitted diseases and AIDS, and common conditions such as pelvic floor disorders [13]. Whereas breast and cervical cancer are among the most fatal causes of death among women. In addition, the increasing number of sexually transmitted diseases and AIDS, which are spreading by high-risk behaviors, can be prevented by promoting related self-care programs. Unwanted pregnancy and subsequent unsafe abortion can also have adverse consequences such as mortality and complications such as infection, infertility, and social disorders, especially for female adolescents, and requires self-care and prevention promotion programs [35]. Besides, nowadays women’s self-care in the reproductive age is suggested as an essential agenda [36]. Screening and prevention consultations can be affordable by increasing the counseling services and employing trained women’s health personnel such as midwives. It seems that providing these services by midwives not only in primary health care centers but also in health houses of the municipality can improve women’s self-care for preventing STIs/HIV, breast and cervix cancers, unwanted pregnancies, and unsafe abortion. Besides, the promotion of self-care behaviors among women for seeking care for periodic breast exams, Pap smears, screening and treatment of sexually transmitted diseases, HIV Rapid testing, and avoiding high-risk sexual behaviors improves women’s SRH [37].

Findings indicated that women’s self-care in physical health is the third priority. It appears women pay more attention to their physical health rather than their psychological or reproductive health. It seems the women care more about their nutrition, exercise and vitamin intake, weight control, and general screening test. But, it should be notable that the average score of self-care in physical health showed still 44 to 50% gaps in the self-care. Therefore, self-care promotion is emphasized in all items of physical health, especially in some items with low scores such as sleep disorders, accident prevention, avoiding tobacco, alcohol, and drugs; the safe use of cell phones and the internet. At the same time, the challenge that exists in the physical health dimension which is an insensitive area of health can be easily overcome by some educational interventions through social marketing and using mass media [38].

The results showed the women’s self-care score of about 70% for screening and periodic testing, but it is still demonstrating a 30% gap to the ideal score. It seems that providing the general screening tests in many primary health care services in Tehran, and the appropriate insurance coverage of the general screening tests have been effective in increasing this rate. Furthermore, Tehran Municipality affords a few health clinics in the city. They can provide laboratory services to women in deprived and high-risk areas and neighborhoods. In addition, the municipality has some agreements with Tehran Universities of Medical Sciences to cooperate with the city’s health centers for follow-up services for women in need of care [39].

The results showed a significant positive correlation between total self-care and women’s education and their husband’s education. These findings demonstrate the positive effect of educational level on health behaviors including self-care, which is confirmed in another study [29]. UNESCO emphasizes education as an essential factor to achieve a healthy and productive life [40]. Since low levels of education are predictive of poor self-care, interventions for improving women’s literacy can promote self-care behaviors among people with low levels of education. Otherwise, illiterate or low-literate people need more education about health and self-care. It can also be attributed to that high-level educated people understand the importance of self-care more than low-level educated individuals.

There was also a significant correlation between the women’s self–care and the family’s income that indicates the impact of financial status on women’s health and self-care. There is no doubt that self-care behaviors come at a cost, and it seems that poor people pay for health expenses, after meeting basic needs such as food, housing, and clothing, and if there are no signs and symptoms of illness, they do not consider health as a basic need and a priority [41]. It seems that the negative correlation between family size and the number of children with self-care behavior can also be due to the effect of these variables on the financial adequacy of the family to meet the basic needs of family members. It should be considered that the deepening trend of the Gini index in recent years may lead to inequality in the health indicators of society [42].

One of the strengths of this study was applying a standard questionnaire that was developed and validated in the first stage of the study. Self-care questionnaire for reproductive-aged women with a validity and reliability index of more than 0.9 was designed based on the recommended care criteria by reputable sources of women’s health institutions such as the American College of Obstetrics and Gynecology. Another strength of this research was a large sample that was taken with normal distribution from all 22 districts and neighborhoods of Tehran.

A limitation of the present study was the focus on women’s health, which was proposed and implemented in terms of the importance of women’s empowerment in health. Besides, the researchers acknowledge that women with disabilities have special health needs and were not considered as the subjects of the study, so further research on disabled women can be considered for future studies. One of the problems of this research was the coincidence of conducting the research with the corona pandemic, which could have led to a change in some health behaviors, such as referring to health centers and hospitals for screening tests, exams, and care services. Besides, we used a multi-stage sampling method. Although the first and second stages of sampling were performed using the random sampling method, the last stage was non-random sampling, and so we suggest random sampling in the last stage in future similar studies.

## Conclusion

The findings showed that reproductive-aged women are performing self-care inadequately in Tehran. The most important challenge in their self-care behaviors was related to mental health and reproductive-sexual health. It seems to provide an interventional package for Promoting women’s self-care in four areas of physical, psychosocial, reproductive-sexual health, and screening tests, with an emphasis on psychosocial and reproductive health, which is necessary for Tehran.

### Supplementary Information

**Additional file 1.**

## Data Availability

All relevant raw data will be freely available to any scientist wishing to use them for non-commercial purposes, without breaching participant confidentiality. The datasets generated and/or analyzed during the current study are not publicly available because sending the data needs obtaining permission from the university but are available from the corresponding author upon reasonable request.

## Abbreviations

WHO: World Health Organization
AIDS: Acquired Immune Deficiency Syndrome
STIs/HIV: sexually transmitted infections/ human immunodeficiency virus
SRH: Sexual Reproductive Health
ACOG: American College of Obstetricians and Gynecologists
RWSCQ: Reproductive-aged Women Health Self Care
WPSI: Women’s Preventive Services Initiatives
CDC: Centers for Disease Control and Prevention
CVI: Content Validity Index
CVR: Content Validity Ratio
UNESCO: The United Nations Educational, Scientific and Cultural Organization
