# Supplementary material for: The self-care situation analysis of reproductive-aged women in Tehran: a survey study
Source: BMC Womens Health. 2023 Nov 25;23:624. doi: 10.1186/s12905-023-02763-9 (PMC10675894; doi:10.1186/s12905-023-02763-9)
Supplement: Supplementary file 1 — Additional file 1. [file 12905_2023_2763_MOESM1_ESM.docx]

**Appendix 1. The questionnaire to assess Reproductive-aged Women's Health Self Care (RWSCQ-36)**

| Yes, I did | Yes, somewhat/ I intend to do | No, I didn’t | Did you perform the following self-cares or consultations during the last few years? | No |
| --- | --- | --- | --- | --- |
|  |  |  | Measurement of height and weight | 1 |
|  |  |  | Nutrition status | 2 |
|  |  |  | Physical activity status | 3 |
|  |  |  | Safety in using the internet and mobile phone | 4 |
|  |  |  | Exposure of the skin to sunlight for absorption of Vitamin D | 5 |
|  |  |  | Examining the state of oral health and consumption of Fluoride | 6 |
|  |  |  | Periodic tests to prevent the risk of chronic diseases (family history, high blood pressure, high cholesterol, obesity, diabetes, and thyroid) | 7 |
|  |  |  | Avoiding alcohol, cigarettes, tobacco, etc. | 8 |
|  |  |  | Intake of vitamins and minerals (especially vitamin D, calcium, and folic acid) | 9 |
|  |  |  | Use of complementary and herbal medicines | 10 |
|  |  |  | Sleep disorders | 11 |
|  |  |  | Lifestyles (exercise, nutrition, sleep, rest, and recreation) | 12 |
|  |  |  | Safe driving (wearing a seat belt, not taking drugs and alcohol). | 13 |
|  |  |  | Prevention of accidents (Sports safety, occupational hazards, hearing protection, etc.) | 14 |
|  |  |  | State of Depression | 15 |
|  |  |  | State of anxiety and stress | 16 |
|  |  |  | The state of thoughts and attempts to commit suicide | 17 |
|  |  |  | Individual and family relationships | 18 |
|  |  |  | Physical, sexual, and emotional violence | 19 |
|  |  |  | Job satisfaction | 20 |
|  |  |  | Observance of menstrual hygiene (use of single-use sanitary pads, and how to wash) | 21 |
|  |  |  | Contraceptives | 22 |
|  |  |  | Breast self-exam | 23 |
|  |  |  | Breast examinations by health personnel in the last 3 years | 24 |
|  |  |  | Cervical screening in the last 3 years | 25 |
|  |  |  | Prevention of sexually transmitted diseases and HIV/AIDS | 26 |
|  |  |  | Screening and treatment of genital infections (chlamydia and gonorrhea) if sexually active | 27 |
|  |  |  | Testing for AIDS if active, if sexually active | 28 |
|  |  |  | Sexual function and disorders | 29 |
|  |  |  | Examination of urinary and fecal incontinence | 30 |
|  |  |  | Investigation of pelvic floor disorders | 31 |
|  |  |  | Mammography (breast imaging) | 32 |
|  |  |  | Blood lipid screening in the last 5 years | 33 |
|  |  |  | Thyroid screening in the last 5 years | 34 |
|  |  |  | Blood lipid screening in the last 5 years | 35 |
|  |  |  | Thyroid screening in the last 5 years | 36 |

**RWSCQ-36:** The questionnaire to assess Reproductive-aged Women's Health Self Care (RWSCQ-36) is a tool to assess the self-care behavior of women in their reproductive age. This questionnaire can be used by researchers or clinicians to identify challenges in the self-care behaviors of reproductive-aged women and to determine the need for interventions to promote self-care in individuals and society.

This questionnaire was developed with 36 items in 4 domains including the physical health domain with 14 items (items 1-14), psychosocial health with 6 items (items 15-20), and reproductive-sexual health with 12 items (items 21-32) and periodic tests with 4 items (items 33-36). The questionnaire assessed the self-care of 19-55 years old women.

**Face validity:** All items of the questionnaire had an item score of more than 1.5 and so were considered important by the participants. The calculated Impact scores of RWSCQ were 2.47 to 4.86.

**The Content Validity:** The content Validity of the questionnaire was assessed by calculating the Content Validity Ratio (CVR) and Content Validity Index (CVI). The results showed CVR ranged from 0.83 to 1. The modified content validity index of I-CVI for all items ranged from 0.91 to 1, and the S-CVI / Ave score was 0.97.

**The Reliability:** The reliability of the questionnaire was measured by calculating Cronbach's alpha coefficient for internal consistency assessment and also calculating the Pearson coefficient to measure the stability of the questionnaire by the test-retest method on 15 reproductive-aged women. The results showed the reliability of the questionnaire by 0.93 for RWSCQ and Intra-class Correlation Coefficient ICC= 0.93.

**The Scoring:** The items were scored 1 to 3 in the responses of “No, I did not”, “Yes, somewhat/ I intend to do” and “Yes I did”, respectively. The score ranges for the different dimensions of the questionnaire including physical-, psychosocial-, and sexual-reproductive health and screening tests were 14 to 42, 6 to 18, 12 to 36, and 4 to 16, respectively, and for the whole questionnaire was 36-108 for RWSCQ-36. The higher scores indicate healthier self-care behaviors of reproductive-aged women. The score of each domain and the total score were calculated and then converted to the standardized 0 to 100 score using the following equation (X-Min Score / Max-Min Score) × 100.
